# Visual analysis of density and velocity profiles in dense 3D granular gases

**DOI:** 10.1038/s41598-021-89949-z

**Published:** 2021-05-19

**Authors:** Dmitry Puzyrev, David Fischer, Kirsten Harth, Torsten Trittel, Raúl Cruz Hidalgo, Eric Falcon, Martial Noirhomme, Eric Opsomer, Nicolas Vandewalle, Yves Garrabos, Carole Lecoutre, Fabien Palencia, Ralf Stannarius

**Affiliations:** 1 Institute of Physics, Otto von Guericke University, 39106 Magdeburg, Germany; 2 Física y Matemática Aplicada, Facultad de Ciencias, Universidad de Navarra, 31080 Pamplona, Spain; 3 Université de Paris, MSC, UMR 7057 CNRS, 75 013 Paris, France; 4 GRASP, Liège Université, 4000 Liège, Belgium; 5 CNRS, Univ. Bordeaux, Bordeaux INP, ICMCB, UMR 5026, 33600 Pessac, France

**Keywords:** Statistical physics, thermodynamics and nonlinear dynamics, Nonlinear phenomena, Imaging techniques

## Abstract

Granular multiparticle ensembles are of interest from fundamental statistical viewpoints as well as for the understanding of collective processes in industry and in nature. Extraction of physical data from optical observations of three-dimensional (3D) granular ensembles poses considerable problems. Particle-based tracking is possible only at low volume fractions, not in clusters. We apply shadow-based and feature-tracking methods to analyze the dynamics of granular gases in a container with vibrating side walls under microgravity. In order to validate the reliability of these optical analysis methods, we perform numerical simulations of ensembles similar to the experiment. The simulation output is graphically rendered to mimic the experimentally obtained images. We validate the output of the optical analysis methods on the basis of this ground truth information. This approach provides insight in two interconnected problems: the confirmation of the accuracy of the simulations and the test of the applicability of the visual analysis. The proposed approach can be used for further investigations of dynamical properties of such media, including the granular Leidenfrost effect, granular cooling, and gas-clustering transitions.

## Introduction

Ensembles of macroscopic, hard particles that interact by inelastic collisions are of considerable academic interest as a test for analytical and numerical treatments of multiparticle systems. Moreover, they are also of fundamental importance, e.g. for the understanding of fluidization of granular materials, of granular flow and behaviour under shear. Dilute ensembles, commonly referred to as granular gases, are relevant for the understanding of the interactions of cosmic dust particles that lead to their self-organization to high-density clusters, planetesimals, large orbs and planetary structures. Similar ensembles of hard macroscopic particles are present in terrestrial natural phenomena as well, e.g. in the form of whirlwinds (dust devils) and avalanches, or in technological processes, e.g. in agriculture, pharmaceutical or construction industry.

The dissipative nature of the particle-particle interactions distinguishes granular gases qualitatively from atomic or molecular gases. A comprehensive introduction to granular gases is given, e.g. in Ref.^1^. We will focus on such systems here, but in principle many of the ideas detailed here may also be exploited for the study of much denser, fluidized granular beds.

Experimental and theoretical investigations of granular gases can be roughly grouped into two classes: The first one deals with granular gases in absence of external forcing, while the second one investigates stationary states under permanent energy entry from the exterior that compensates the dissipative losses of kinetic energy. The investigation of the former scenario, granular cooling of granular gases^2^, has attracted considerable theoretical efforts, while experimental studies remained comparably scarce. Experiments were so far largely restricted to small ensembles ($<1000$ particles) and comparably short observation times where the particle number density in the observed volume remained roughly uniform ^3–10^. At long time scales, the uniform distribution is unstable and one expects the formation of spatial inhomogenities and eventually of clusters^11–19^. This appears to be one of the preconditions for the formation of cosmic objects from more or less uniformly distributed microscopic dust^20^.

A second class of studies considers granular gases under constant or periodic energy supply from outside. This can be achieved, for example, in a 3D volume by mechanical vibration of container walls ^21–29^, or of the complete container^30–33^. One may also excite the particles in the volume directly with non-stationary random or periodic magnetic fields^34–36^. Such experiments are less susceptible to the effects of gravitation, so that some studies can be performed in the low-quality microgravity environment of parabolic flights ^26–28,30–34^ or even under normal gravity ^22,37–42^. The interesting aspects of such experiments are, among others, the distribution of mean kinetic energies among the individual degrees of freedom of the particles, the velocity distribution functions, and the spatial distribution of the particles in the observed volume.

One of the activities to study granular gases under strong excitation is the *Space Grains* project^43^. Within this cooperative effort of several research groups, successful experiments have been performed on parabolic flights, and an ISS mission is being prepared. Among several aims^43^, one is the study of clustering transitions of a granular material excited in a cuboid box by two pistons on opposing sides in anti-phase^27,28^. Experimental activities are accompanied by comprehensive numerical efforts^44–46^. One of the interesting features is the formation of a zone of densely assembled ’cold’ (low kinetic energy) particles in the central part of the observed volume, which is flanked by two regions with ’hot’ (high kinetic energy) particles of lower particle number densities towards the excitation pistons. Eshuis et al.^37^ described a similar phenomenon in shaken granular matter in normal gravity as a granular analogue of the Leidenfrost effect, where a liquid droplet levitates above a hot plate on a vapor layer. A similar situation occurs when the pistons move in phase, i.e. conserving the volume available to the grains^30,32^. Menendez et al.^47^ studied this ’granular Leidenfrost effect’ in microgravity using X-ray imaging. They established a characteristic state diagram with the excitation strength and the filling fraction as its essential parameters. Qualitatively similar results were reported under (non volume-conserving) vibrational in-phase excitation^27,28^. Most of these experiments were performed with spherical particles, with the advantage that numerical simulations of collisions are particularly simple. Often, they are even approximated by using a constant normal restitution only.

Here, we extend this study to cylindrical particles. There are strong arguments to use cylinders instead of spheres: The number of particle-particle collisions at a given particle number density and particle volume is considerably larger for elongated cylinders than for spheres^5,24,26^. Thus, one finds more inter-particle collisions in a given ensemble but approximately the same number of particle-wall collisions. In low filling fraction experiments, this is advantageous since the latter disturb the bulk dynamics and tamper the behaviour of the whole ensemble. With elongated cylinders, one can perform experiments with the same number of particle-particle collisions at lower particle number densities, which is favorable for the optical observation of the ensemble (less coverage of particles in the back). An equally interesting aspect is that the cylinders add a rich variety of effects to the collision processes^24,48–50^. For example, rotational degrees of freedom can be excited without frictional contacts between the cylinders. Collisions with plane container walls are more complex than for spheres^48,51^. It is much easier than for spheres to identify the rotational degrees of freedom of the particles and to evaluate rotational kinetic energies.

A key problem of the quantitative analysis is the determination of particle positions, velocities and orientations from optical video footage. In general, this includes the problem of 3D tracking of particles on the basis of two stereoscopic images, which is particularly challenging because of particle overlaps, see, e.g., the experimental data in Refs.^5,25,52^. This demanding task can be tackled manually^25^ or using Machine Learning algorithms^53^, but even 2D tracking becomes complicated when the filling fraction of the observed volume is more than a few percent, because particles in front screen background particles. Particle assignment in subsequent videos is not always trivial. Shadows cast by the grains or reflections on the particle surfaces make a homogeneous illumination practically impossible. Sophisticated tracking mechanisms for clouds of spherical particles at low filling fractions were recently presented with application to dusty plasmas^54^. Some information on the evolution of a sufficiently dilute granular ensemble, the local filling fraction and general trends of the direction of motion can be obtained by averaging image sequences^26^. Even accurate information on particle distributions may be derived from reflection patterns in a granular gas of spheres^6^, a method not straightforwardly extendable to elongated or irregular grains. Particularly in local regions of high filling fractions, methods evaluating the optical density of the granular ensemble from a 2D perspective suffer from particle overlaps. They essentially reflect the behavior of the front particles, while little information is available on the grains behind. Similar challenges of 3D and 2D tracking of multiple particles are often encountered in the studies of complex (dusty) plasmas^54,55^.

For the spherical grains, Kolmogorov-Smirnov (KS) tests provide a solid criterion to distinguish between a gaseous state and a dynamic cluster of particles ^45^. The method was successfully applied in Refs.^28,56^. For elongated grain shapes, however, the situation is much more complex and KS-tests tend to fail. Several conditions for this method are violated: Because of the multiple possible 3D orientations, elongated particles cannot be separated from each other by dividing the container into equally thick slices as it can be done for spheres. Moreover, the number of independent slices (classes) needed to perform a reliable KS-test cannot be attained. This problem increases with higher aspect ratios of the grains.

The aim of this study is to develop methods to extract local velocity, kinetic energy, and particle number density data from densely packed, periodically excited granular ensembles of cylindrical rods. We apply this approach to synthetic data to test the correctness and reliability of the mathematical analysis, and further apply the method to experimental data from parabolic flights. The approach is equally suited to solve the simpler problem of ensembles of spherical particles.

## Experimental setup

**Figure 1:**
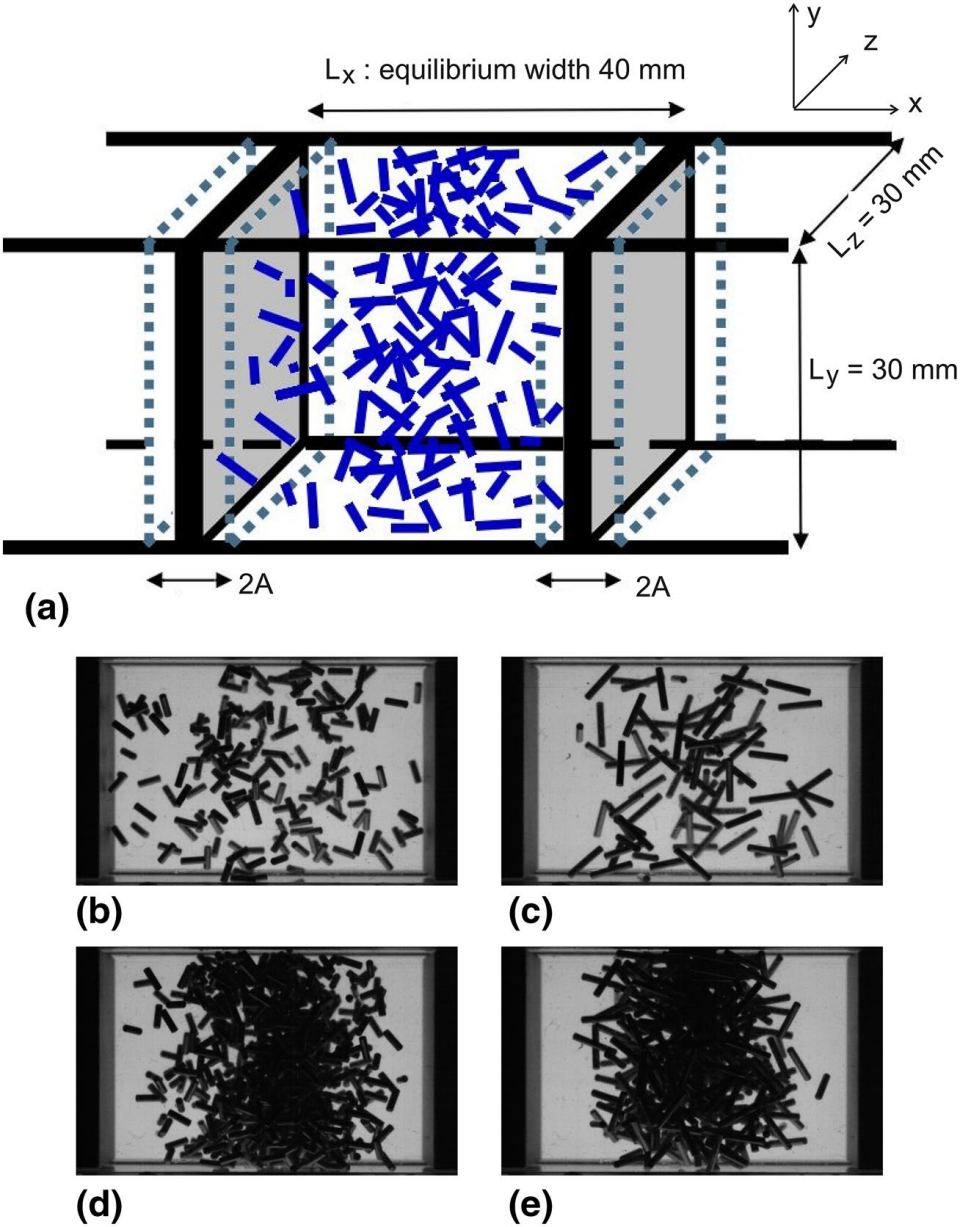
(**a**) Sketch of the relevant parts of the experimental setup (for details see Ref.^43^). Images are recorded from the front and the bottom with respective illumination from the back and the top. The grey side walls can be vibrated by actuating voice coils. (**b**–**e**) Exemplary experimental snapshots of the bottom view showing (**b**) 183 rods of $\ell =3$ mm length, (**c**) 92 rods of $\ell =6$ mm length (both corresponding to a filling fraction $\phi =1\%$), (**d**) 733 rods, $\ell =3$ mm and (**e**) 367 rods, $\ell = 6$ mm (both with $\phi =4\%$).

Experiments were performed with the Vip-Gran instrument during two ESA parabolic flight campaigns (PFC 71, May 21–23, 2019, and PFC 72, November 26–28, 2019 at Novespace, Mérignac, France). A detailed description of the setup and experiments is found in Ref.^43^. The parabolic flight equipment was built by DTM Technologies (Modena, Italy). Here, only few aspects are important: the type and parameters of excitation, the shape, size and amount of granular material, and the imaging method. Figure 1 shows a sketch of the container and selected examples of experimental images. The container possesses a square cross-section of $30\times 30~{\text{{mm}}}^2$, and two opposing movable side walls (pistons) with a mean distance $L=40~{\text{{mm}}}$ at rest. Agitation of the system is realized by two voice coils vibrating these pistons horizontally with an amplitude $A=4$ mm at a frequency of 15 Hz in the experiments described here. The maximum acceleration of the plates is thus $\approx 3.6$ g.

In each run of the experiments, an ensemble of *N* monodisperse steel rods with diameter $d=1~{\text{{mm}}}$ and lengths between $\ell =3~{\text{{mm}}}$ and $\ell =10~{\text{{mm}}}$ are enclosed in a cuboid container (the cylinder ends are slightly domed so that the overall lengths are 0.5 mm longer than the cylinder barrel). Between individual runs, the number of particles was systematically increased, e. g., from 180 to 1100 for the 3 mm long rods, covering mean filling fractions $\phi =(N\pi d^2 \ell )/(4 L_x L_y L_z)$ between $1~\%$ and $6~\%$ inside the cell. The filling fraction changes during the piston movement by $\pm 20$ % as the cell volume is not conserved. We refer here to the mean filling fraction $\phi$ for a piston separation of $L=40$ mm.

Bottom and front sides of the container are illuminated by two LED panels. The grain ensemble seen from these perpendicular perspectives is recorded at a frame rate of 900 pictures per second by two synchronized high speed cameras (*Mikrotron EoSens 4CXP*) at a resolution of $1024\times 900$ pixels (20 px/mm).

## Visual experimental data analysis

In the following, we describe and assess methods for extracting the averaged density profiles, as well as the velocity or energy profiles for a fixed phase of the excitation. This assumes that the granular gas is in a steady state in the way that the overall dynamics periodically repeat in consecutive periods of the excitation, so that data corresponding to the same phase can be averaged.

### General approach

An image analysis for spheres providing local particle number densities in similar experiments was successfully employed earlier. The introduction of the analysis methods is given for beads (spherical particles) in Refs.^28,45,56^. The method’s viability was indirectly confirmed by the comparison of results of a visual analysis of the experiment to numerical simulations with regard to gas-clustering transitions. Namely, the threshold obtained by the Kolmogorov–Smirnov test (at given confidence level) of density profiles obtained from experimental data roughly corresponds to the threshold found by studying the caging threshold in the simulated data.

Here, we perform a more thorough analysis of the shadow density profile for elongated particles. Furthermore, we suggest a method based on feature tracking to extract the mean velocity profile from image sequences.

Our approach is based on the following methodology: Numerical simulation provides particle positions and orientations as well as velocities for a given ensemble, using realistic simulation parameters. ’Synthetic’ images are created from these ensemble data. These images are analyzed with the proposed evaluation tools and compared with data extracted with the same tools from experimental images. While the individual particle arrangements will naturally differ in experiment and simulation, we take agreement between the statistical ensemble data as the decisive criterion to test both the reliability of the simulation and the accuracy of the evaluation approach. We can presuppose that the possibility of both methods giving a similar but incorrect profile which mimics the observed images naturally exists, but is minimal. This approach allows us to infer a reasonable degree of reliability both to the simulation technique and to the visual analysis method. We do not aim at a detailed agreement between experiment and simulation in all physical statistical quantities here, such a first study is found in Ref.^57^ for the experiment from Ref.^24^ using similar code to what is employed here.

We used a hybrid GPU-CPU implementation of discrete element modelling (DEM)^49,58^. This numerical tool was adapted to simulate confined systems with moving walls. The model considers the evolving dynamics of a monodisperse ensemble of spherocylinders, with length $\ell$ and radius *r*, i.e. aspect ratio $\zeta =\ell /2r$. The contact detection between two spherocylinders reduces to finding the closest point between two line segments. Thus, the overlap distance $\delta$ simply results from the overlap of two spheres of radius *r*. For the particle-wall collisions, we used the same model but assuming the interaction of a spherocylinder with an infinite moving plane. The force $\vec {F}_{ij}$ exerted on particle *i* by the particle *j* reads as: $\vec {F}_{ij} = - \vec {F}_{ji}$ , and it can be decomposed as $\vec {F}_{ij} = F^{\text{n}}\cdot \vec {n} + F^{\text{t}}\cdot \vec {t},$ where $F^{\text{n}}$ is the component normal to the contact plane and $F^{\text{t}}$ acts in the tangential direction. Here, $F^{\text{n}}$ was a Hertz-type force^59^, depending on the overlap distance $\delta$ between two spherocylinders. For the sake of simplicity, we assumed frictionless particles $F^{\text{t}}=0$.

The simulated container was defined with the same geometry as in the experiment, in the same coordinate system. Similar to the experiment, an amplitude $$A = 4$$ mm, a frequency $f = 15$ Hz, and a mean distance $$L=40\,$$mm were chosen. Besides, to mimic the behavior of steel particles, we have used a particle density $$\rho _p = 7850$$ kg/m$$^3$$ and a Young modulus $$Y = 1~{\text{{GPa}}}$$. For the container walls, we have used a Young modulus $$Y_w = 6~{\text{{GPa}}}$$. The energy loss was quantified using an effective restitution coefficient $$e_n = 0.8$$, from which the collision damping parameter of the model was obtained, $$\beta =\frac{\ln {e_n}}{\sqrt{\ln {e_n}^2+\pi ^2}}$$^60^. A simulation time step was chosen equal to $$\delta t = 5/90~ \upmu$$s^61^.

We concentrate here mainly on a part of the data set (of the 71. and 72. ESA PFC) with cylindrical rods of aspect ratio 3. This choice of short rods minimizes the effects of particle rotations on the evaluation approach (see below), which may require further refinement of the analysis. Note that every method successful for short cylindrical rods will also be suitable for spheres. The influence of the rotational degrees of freedom would vanish in ensembles of spherical grains.

### Confirmation of the density profile analysis for elongated cylinders

**Figure 2:**
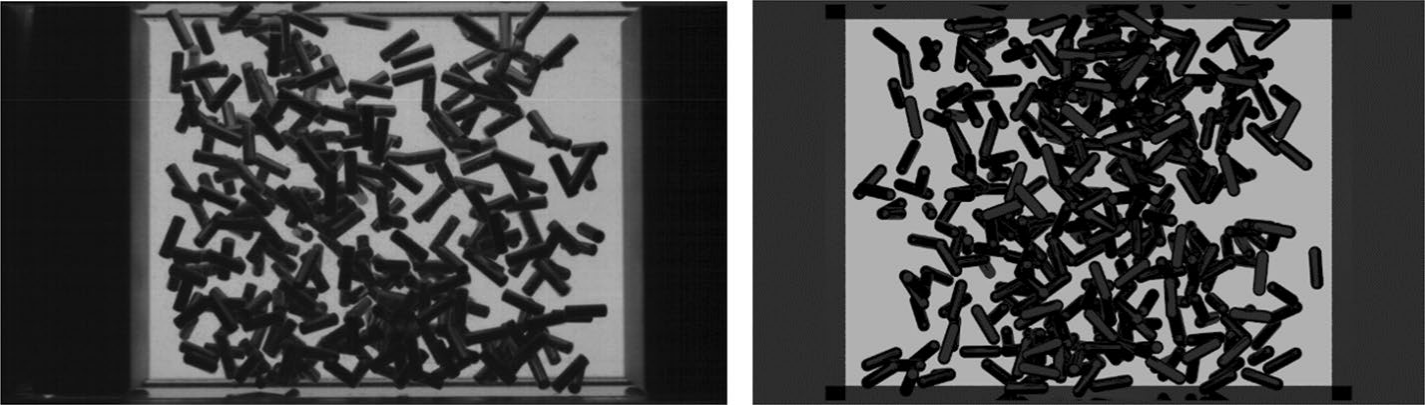
Left: video snapshot from the experiment for $N=367$ rods (filling fraction $\phi =2.0\%$). Right: render of a simulated frame with the same number of rods. The size of the field of view is $40\times 30$ mm$^2$.

We performed simulations for 183, 367 and 733 spherocylindrical particles of 3 mm length and 1 mm diameter with parameters similar to the experiment. Namely, we were aiming to simulate the 1st, 6th and 16th parabolas of 1st day, ESA PFC71. First, 10,000 simulated frames with the same frame rate as in the experiment were created (corresponding to $\approx 11$ s of the 900 fps video), see Fig. 2. The time steps between the simulated frames equal those in the experiment. Resolution, contrast and brightness of the synthetic images were calibrated to adequately reproduce the features of the experimental videos.

**Figure 3:**
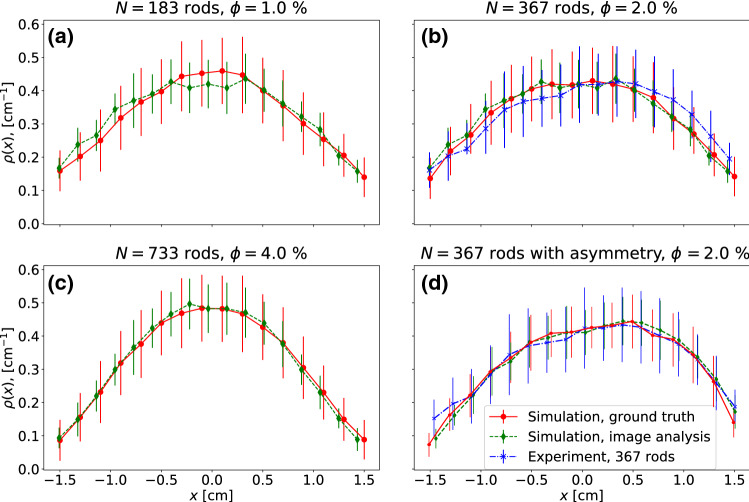
Normalized density profiles from a simulation of (**a**) 183, (**b**) 367 and (**c**) 733 rods, ground truth compared to the profile obtained using the shadow density analysis from both front and bottom perspective views. A density profile obtained with the same method from the experimental videos for 367 rods is shown in (**d**). Results are averaged over 100 periods in the simulation and 50 periods in the experiment, for the middle position of the pistons ($L=40~{\text{mm}}$), moving outward (corresponds to frame number 45 in Fig. 4).

We then extracted the density profiles along the horizontal axis (following Ref.^28^) for both experimental and simulated data. First, a brightness threshold was applied to the image to separate shadowed areas from illuminated ones. Then, the image was separated into slices with the length of an effective particle diameter $d$ along the horizontal axis. From the ratio of shadowed to illuminated areas, the approximate number of particles in each slice can be inferred. The effective particle diameter was chosen as $d'=34$ pixel (corresponding to $1.7~{\text{{mm}}}$), roughly estimated from the average single particle shadow area. Figure 3 shows the ensemble-averaged density profiles for a given piston position ($L = 40~{\text{{mm}}}$, pistons moving outward). Following Refs.^27,28,56^, the local density $\rho (x)$ is calculated between the closest piston positions $-L/2+A<x<L/2-A$ and normalized so that the integral of $\rho (x)$ equals 1. We calculated the density profiles from the true particle positions in the numerically simulated data (*ground truth*), from the shadow density of corresponding simulated images, and from the shadow density of experimental video frames. Both perspective views (front and bottom) were used for shadow density analysis. The density profiles were averaged over 100 periods for simulation and 50 periods for the experiment (the periods of high-quality microgravity in the respective parabolas were relatively short). Here we use video footage from frame 3800 to frame 6800. The plot includes error bars for the standard deviation.

**Figure 4:**
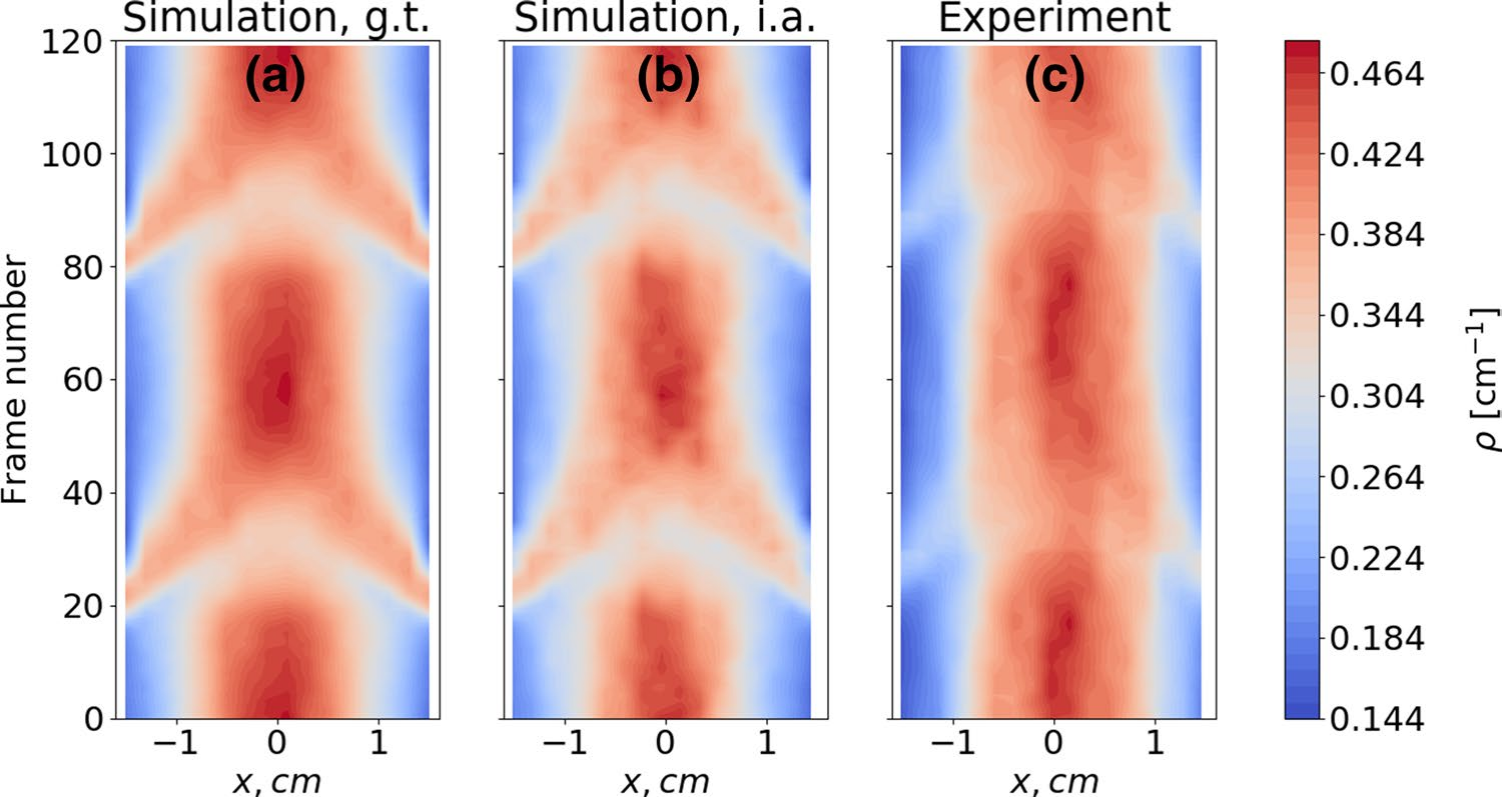
Normalized density profiles for $N=367$ rods, the filling fraction is $\phi =2.0~\%$. (**a**) Ground truth from the simulation, (**b**) reconstruction from the synthetic images of simulated data, (**c**) reconstruction using the experimental videos from one of the parabolic flight experiments. Data were obtained by synchronous sampling across 100 periods in the simulation and 50 periods in the experiment.

We observe in Fig. 3 that the ground truth average density profile from the simulation is reasonably well approximated by the shadow profile analysis method for various packing fractions, see Fig. 3a–c. This proves the applicability of the evaluation approach. Figure 4 shows space-time plots during two periods of piston vibration, extracted from the simulation of 367 rods, as well as one of the parabolic flight experiments. The time axis (frame numbers) runs from bottom to top. We compare the ground truth density profiles (Fig. 4a) with the ones obtained from the analysis of synthetic images (Fig. 4b), and with the result of the analysis of experimental video footage (Fig. 4c) for the 6th parabola of day 1, ESA PFC71. All three space-time plots show satisfactory agreement. The largest discrepancy is observed around frame numbers 20–40, where the influx of the particles from the vibrating walls to the center of the container is observed. Apparently, the main reason is that our simulation of frictionless particles is not yet fine-tuned to accurately reproduce the experiment with frictional grains. While these simulations generally reproduce the density profiles quite well, some dynamical effects are not accurately captured. Namely, the particles in the experiment gather more robustly near the center of the container in comparison to the simulations, which could be an effect of additional dissipation due to particle-particle friction.

Additionally, a slight asymmetry of the density profile was observed in the visual analysis of the experimental data, see Figs. 3d and 4c. It can be attributed to *g*-jitter (residual acceleration) in the *x*-direction which reaches values of 0.005 g for this particular time frame of the PFC71 parabolic flight. It is worthwhile to analyse if such a shape of the profile can be reconstructed with the shadow density method. We have performed additional simulations, where a slight variation between left and right wall collision parameters was artificially introduced. This resulted in an asymmetry of the simulated density profile that resembles the one observed in the experiment. It turns out that such a profile asymmetry is accurately reproduced by the shadow profile image analysis, see Fig. 3d.

An extensive effort will be required to improve the accuracy of the shadow density analysis for different numbers of particles (filling fractions) and other parameters. However, for the present purpose, we notice that the shapes of the density profile are satisfactorily similar for both simulation and experimental data. A more detailed analysis of experimental data will be possible when more data sets for different parabolas will be available and can be compared with simulations.

### Mean velocity profiles extraction

The density profiles yield some insight to the particle distribution in space, but do not lead to a deeper understanding of their underlying motion. The extraction of mean velocity profiles from experimental data could provide greater insight into the particle dynamics, particularly in regions of different local particle number densities, and will help to detect dynamic clusters^45^. A procedure which at least indirectly allows to infer mean velocity profiles from the time evolution of density profiles was recently suggested^47^. However, this method does not provide information on particle dynamics.

**Figure 5:**
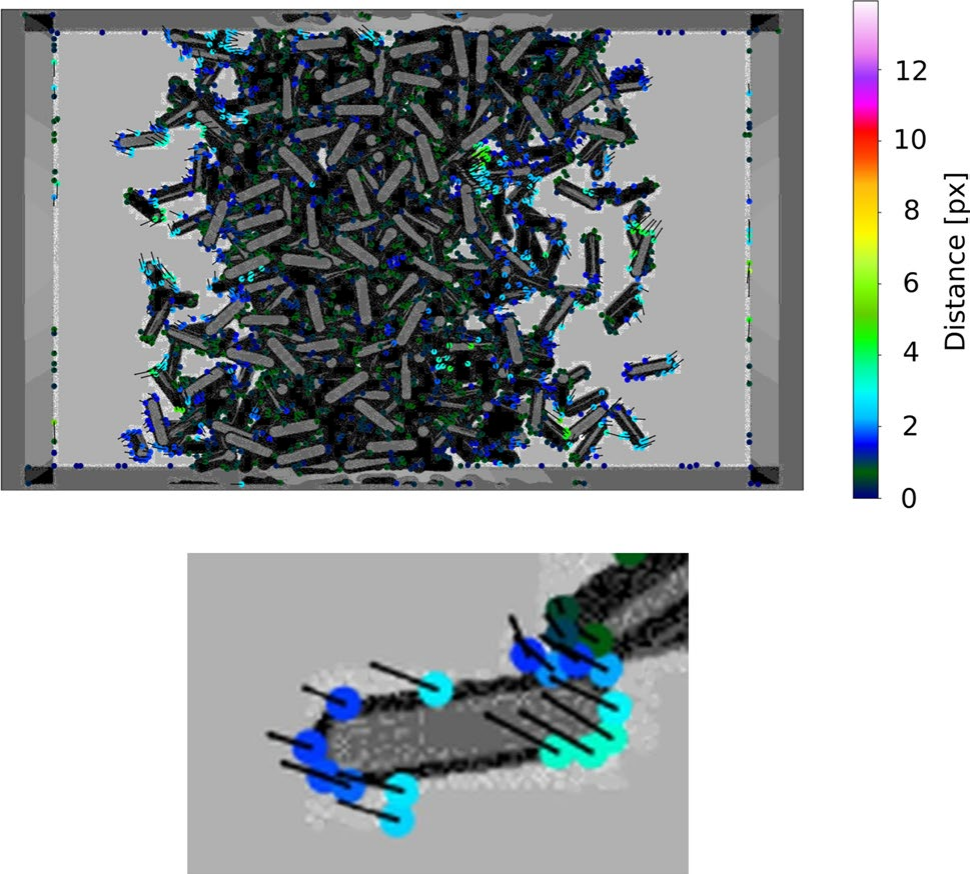
Example of feature displacement detection from two consecutive frames (visualization by the PyTrx package). Small black lines show the directions of feature displacement vectors, while dot colors correspond to the distance (lengths of the displacement) in pixel. The first frame serves as a background. The bottom image is a zoomed-in detail, showing one of the particles and the detected displacements.

In what follows, we describe a method to extract particle velocity profiles which is suitable for the case when most of the individual particles cannot be tracked. We adopted a variation of a PIV (Particle Image Velocimetry) method developed for the glaciological analysis of visual data (estimation of surface ice velocity from time-lapse images). Those methods were realized in the Python software package PyTrx^62^ and applied to the extraction of data in a number of publications^63,64^. Our system is, naturally, very different from the original application scenarios of PyTrx. The visual velocities of glacial profiles are usually comparably slow and variations between the consecutive video frames are very subtle (sometimes subpixel feature movements are analysed). In our case, however, the periodic nature of the experiment allows to average data across many cycles to obtain smoother results. We found that the general approach is feasible after averaging 100–200 cycles, depending on the number of particles in the system. There are two velocity detection procedures in PyTrx, dense (calcDenseVelocity) and sparse (calcSparseVelocity). In the dense mode, frames are separated into small rectangular regions (windows) and a displacement vector is obtained for every window by cross-correlation between corresponding windows in consecutive video frames. In the sparse mode, the Lucas-Kanade optical flow algorithm^65^ is applied using the OpenCV function calcOpticalFlowPyrLK^66^ to find the pairs of corresponding points in a couple of consecutive video frames. For a relatively dilute system like the granular gas, where neighboring particles often move in different directions, the sparse algorithm should perform better. To confirm this, we applied both dense and sparse velocity detection procedures and obtained much more accurate results in the sparse mode.

There are two important parameters in the calcSparseVelocity procedure of the PyTrx package which extracts the velocities from a couple of consecutive frames. One parameter is the **window size**, which governs the maximal search distance between similar features in two frames. The second parameter is the **backtracking threshold**. It works as follows: The features are tracked from the original image to the next. Then the order of the frames is reversed and the features from the second frame are tracked back to the original. Only the points which appear closer to their origin than a threshold value are selected. There is as well a Boolean switch which regulates if image histogram equalization should be performed before performing edge detection or not. We found that to obtain a more accurate reconstruction of the velocity profiles, histogram equalization should be omitted: Turning it on generally allows to detect more feature displacements between subsequent frames, but introduces a lot of noise in extracted velocity data.

**Figure 6:**
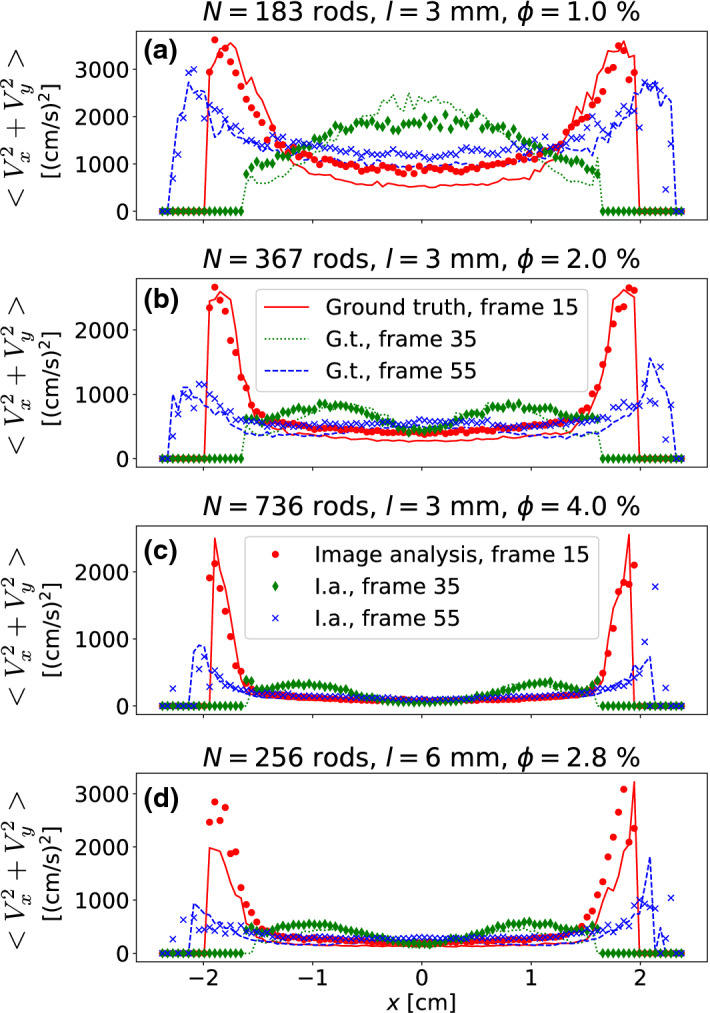
Calibration of mean squared velocity (kinetic energy) profiles along the coordinate *x* on the basis of the evaluation of synthetic images. The lines represent the *ground truth*, i. e. the actual mean squared velocities of the simulated ensemble. Symbols show the reconstructed distributions from synthetic images for different piston positions. Note that some fringe points in the reconstructed data may correspond to tracked features of moving pistons; those data points lie outside the momentary container volume and should be discarded in the subsequent analysis.

We performed a comprehensive search for the best combination of the two parameters mentioned above (window size and backtracking threshold) that provides the most accurate reproduction of the ground truth mean velocity data for two different simulated packing fractions (number of particles). We found that the best results correspond to a window size equal to 30 pixels and a backtracking threshold of 10 pixels. The value set for the window size parameter roughly corresponds to the optical translation of the fastest particles between consecutive frames. Due to the relatively high velocities of particles (in comparison to the frame rate of the video) and visual noise, only few features which travel not too far between subsequent frames and which are not obscured can be accurately backtracked. Consequently, the value for the backtracking threshold has to be relatively large in order to account for particles with higher velocity. Figure 5 shows an example of feature extraction from two consecutive frames, visualized with the PyTrx package. One observes that near the center of the container, most feature displacements are small and nearly uniformly directed. Closer to the vibrating walls, displacements are larger and the ones corresponding to the same rods are often similarly aligned (see the bottom image of Fig. 5 for a zoomed-in detail on one of the particles). This corresponds qualitatively to the expected velocity profiles.

The lines in Fig. 6 denote the mean squared velocity profiles calculated directly from the simulation data, while the markers in the same figure show corresponding graphs reconstructed from the synthetic images of these data. The profiles were averaged for 200 excitation periods. Data are plotted for three selected phases of the piston positions. Frames numbered 15, 35, and 55 in each period of 60 frames correspond to phases of 90$^\circ$, 210$^\circ$ and 330$^\circ$ of the pistons respective to their widest separation. They are denoted by different line and marker styles. Since the mass of each particle is the same and thus not relevant for the calibration procedure, we refer for simplicity to the mean squared velocity in (cm/s)$^2$ as a measure of the mean (translational) kinetic energy $E_T=E_x+E_y$. Note that in the calibration procedure, we consider only two degrees of freedom that correspond to translational motion in the image plane.

**Figure 7:**
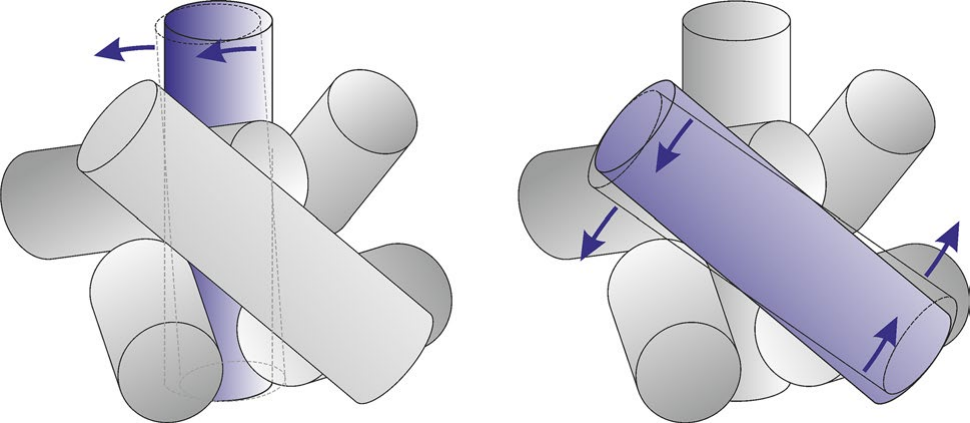
Rotations of rods about their perpendicular axes can be misinterpreted by the evaluation procedure as translational motions, as sketched here. The rod in the background does only rotate, but the detected edge mimics an apparent translational motion to the left. The rotating rod in the foreground has features moving in opposite directions.

One obtains sufficiently good reconstruction results both for the lower and the higher packing fractions of the short $\ell =3~{\text{{mm}}}$ rods, see Fig. 6a–c. For comparison, Fig. 6d shows the result for longer rods of $\ell =6~{\text{{mm}}}$, where the ground truth profile is reconstructed significantly worse. The method overestimates the mean translational kinetic energy for long rods. This is explained by the fact that with an increasing aspect ratio, the contributions of particle rotations to the detected feature velocities increases. At the current state, we cannot reliably separate the rotational and translational degrees of freedom without further assumptions on the kinetics. This is demonstrated in Fig. 7. Rotating grains have features that move in opposite directions even if their centers of mass are at rest. When such particles are partially obscured, the motion of one edge may be misinterpreted as a directed translational motion. This effect becomes more pronounced when the aspect ratio of the particles increases. This limits the accuracy of our method when applied to particles with larger aspect ratios.

**Figure 8:**
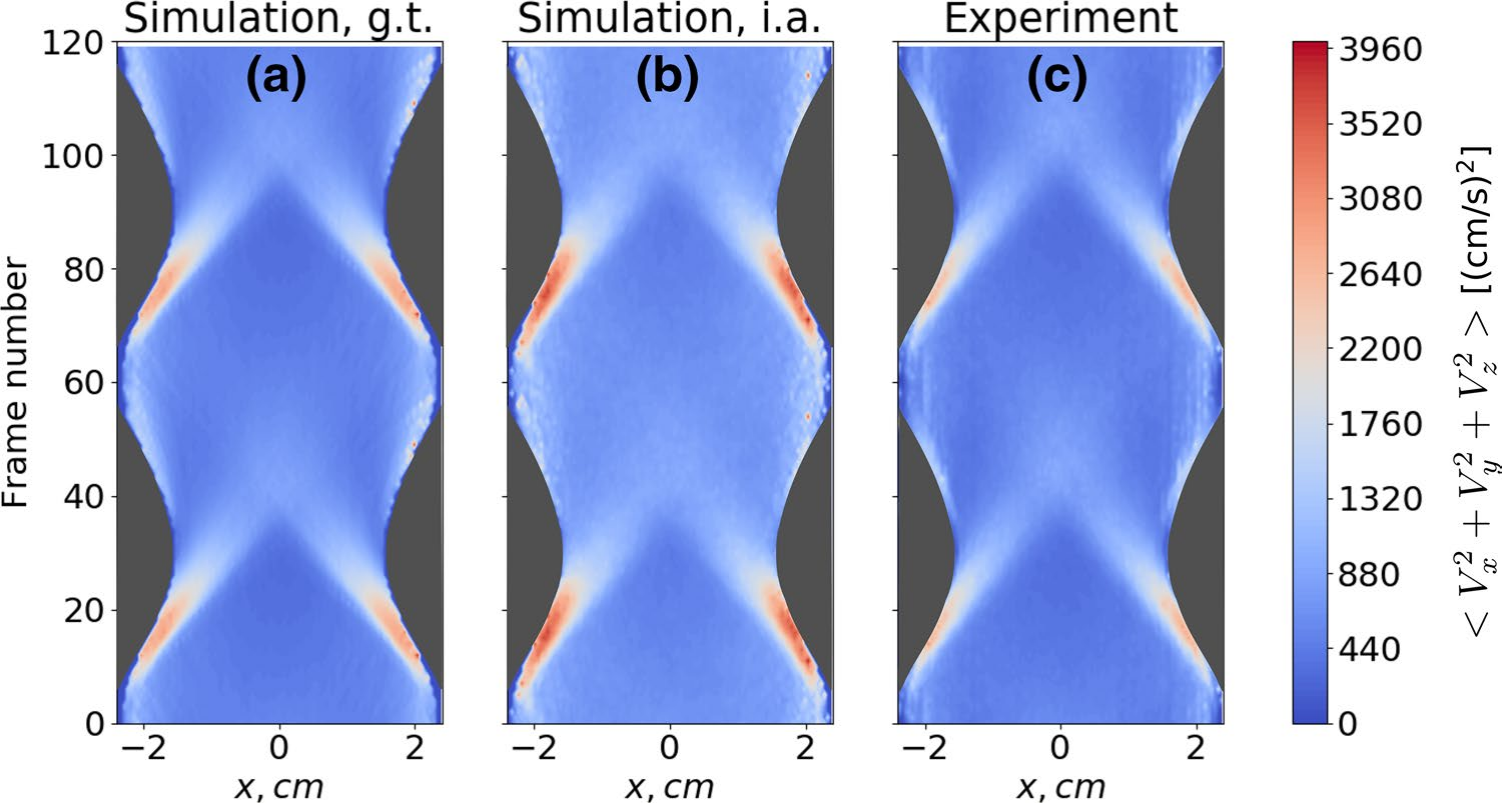
Mean squared velocity profiles for $N=367$ rods, the filling fraction is $\phi =2.0~\%$. (**a**) Ground truth from the simulation, (**b**) reconstruction from the synthetic images of simulated data, (**c**) reconstruction using the experimental video from one of the parabolic flight experiments. Data were obtained by synchronous sampling over 200 cycles of the excitation. Dark gray regions correspond to the areas occupied by the pistons.

After performing the calibration procedure with synthetic data, we demonstrate how the method can be applied to reconstruct mean kinetic energy profiles from the experimental video footage. Figure 8 shows the squared velocity (energy) profiles $E_T=E_x+E_y+E_z$ during two periods of piston vibration (averaged over 200 periods), extracted from one of the parabolic flight experiments. Here, the mean squared velocity in horizontal direction $<V_x^2>$ was averaged from both perspective views, while each of the components $<V_y^2>$ and $<V_z^2>$ was extracted from the one of the corresponding views. The vibration of the two pistons (dark grey regions) at the sides can be identified easily. The time axis (frame numbers) runs from bottom to top. We compare the ground truth energy profiles (Fig. 8a) with the ones obtained from the analysis of synthetic images (Fig. 8b) and with the result of the analysis of experimental video footage (Fig. 8c). All three space-time plots show satisfactory agreement. The influx of particles from the walls seems to be slightly weaker in the experiments compared to the simulation. This can be attributed to shortcomings of the simulation and matches with the discrepancies in the density profiles in Fig. 4, which were discussed above. We note that these results are also in good agreement with the simulated velocity profiles for spherical particles which were shown in Ref.^45^ as well as qualitative experimental results from^47^. A comprehensive study of mean velocity profiles for various microgravity experiments is part of ongoing research.

## Discussion and outlook

### Interpretation of the results

At this point, it is appropriate and necessary to discuss the averaging process and the meaning of reconstructed velocity and energy data from the previous section: Important parameters for the description of the granular gas are the local granular temperature and the local material transport. In our evaluation, we have employed a synchronous averaging over fixed phases of a periodically excited system, assuming that the state of the granular gas is also periodic. We have presented the averaged *squared velocities* in Figs. 6 and 8. If one is interested in the transport processes, one has to average the three velocity components in each position separately. This can be done in the same way as for the squared velocities, and one can construct analogous plots as those shown here. If one is interested in the local granular temperatures, it is necessary to subtract the average local velocity before squaring. This yields the local fluctuations of particle velocities. In order to obtain this quantity, which is one key feature to characterize the dynamic clusters, it is necessary to subtract the square of the local averaged velocities from the local averaged square velocities. As the purpose of this paper was the introduction of an observation and analysis technique, the physical interpretation of these data and the discussion of the ensemble characteristics is beyond its scope. It requires a more detailed discussion of the physical background and will be published in a subsequent paper.

### Main conclusions

The primary purpose of this study is to propose, test and calibrate methods for the characterization of grain ensembles in situations where individual particle tracking is impossible. Its focus is the extraction of information on local packing fractions and mean velocities on the basis of 2D optical images. Even though we have access to two stereoscopic projections of the ensemble, we did not make attempts to extract 3D particle positions or velocity data. This requires the necessity to collate individual particles in both perspectives, which is practically impossible in view of multiple overlaps.

For the present type of granular ensembles, no other evaluation method is available. Thus, we had to design a calibration strategy that uses numerically simulated data. The test procedure is based upon the composition of synthetic frame sequences from simulated particle ensembles, where all information regarding the particles is known for each frame, followed by a subsequent comparison of particle position and velocity data from image analysis with the original data (ground truth).

The shadow density analysis method^28^ yields satisfactory reconstructions of the number density profiles, but it is unsuitable for an extraction of velocities and kinetic energies as key figures of merit in cluster formation. For this purpose, we applied a software (PyTrx) developed for geophysical research^62^ that is based on a feature tracking algorithm. We demonstrated by comparison of velocity data extracted from synthetic images with the original data that this method yields very good results for elongated cylinders or spherocylinders with sufficiently short aspect ratios ($\ell /d<5$). The approach has some adjustable parameters that need to be calibrated for optimal accuracy. This was achieved here with synthetic images and comparison of the original and reconstructed data.

When the aspect ratio of individual grains gets larger, the method is subject to artifacts. It cannot clearly distinguish between rotational and translational motion, particularly of partially masked objects in the rear. Thus, when velocities of detected features are interpreted as translational motion, this shortcoming leads to some overestimation of the translational kinetic energy, demonstrated here for rods with an aspect ratio of 6.

It is also worth noting that with our method, which is based on an optical observation of opaque objects, all kinetic information is sampled from particles near the front of the ensemble. If the system is dense, the kinetics of the rear particles is completely hidden and one needs some model assumptions to make general statements. The method cannot replace non-invasive tomographic imaging techniques^67^, but those require considerably more efforts and they are not available at present with sufficiently short exposure times. X-ray radiography can at least pick up information from all particles including those hidden optically, but since it samples an integral signal, direct conclusions on individual particle kinetics cannot be drawn either.

### Applicability of the visual analysis methods to open questions in granular gas dynamics

One of the main goals of our investigation was to provide a base for solving a variety of open questions in dense granular dynamics, such as gas-clustering transitions^27,28,43–45^, the granular Leidenfrost effect^37,47^ and others. As stated before, shadow-based analysis can be employed for an extraction of density profiles. We have confirmed here that such density profile extraction works well for anisotropic particles. The feasibility for other particle shapes still needs to be checked. The feature tracking analysis for the determination of local velocities and kinetic energies was successfully demonstrated here. This combination of visual analyses provides valuable insights into the system’s structure and dynamics. However, a substantial amount of data is still impossible to reconstruct, due to the nature of the experiment and in particular of the observation techniques. Because of the high packing fractions, most particles are usually obscured. This emphasizes the necessity to accompany such experimental studies by numerical simulations. A by-product of this project was the successful demonstration of numerical simulations that generally reproduce the experimental features qualitatively as well as quantitatively. The density and velocity profile analysis revealed some shortcomings of the simulation technique which may be improved with a refined model. On the basis of the density and velocity data and their combination, it will be possible to search for novel, generalized criteria describing dynamic clustering and phase transitions.

A phenomenon that was studied so far only scarcely is the granular Leidenfrost effect in microgravity^47^. The analysis of this phenomenon with the optical approaches introduced here can provide complementary insights to the integral information obtained from X-ray imaging.

### Outlook

The approach proposed here for the extraction of velocity and kinetic energy data will not only work for short cylinders. It will be equally reliable for spherical particles or any other grains, even irregularly shaped and polydisperse ones like sand, because it does not presuppose a specific grain shape but focuses on suitable features and details in the optical images. This advantageous characteristics is evident from the fact that the algorithm was developed for a situation where the investigated entities are irregular and polydisperse, too.

This implies that the method is applicable for a broad variety of granular systems and a large number of open problems in the physics of granular gases or fluidized granular beds.

Further improvements of this method include the optimization of the illumination technique (front illumination) in order to get more and better features from the images. Coloring particles individually will improve the feature detection and tracking process enormously. The detection and analysis of features is certainly not limited to optical images in the visible spectrum. One can also adapt the method to the analysis of X-ray radiography provided that the particles themselves are resolved. Then, the problem of overlaps becomes less restrictive since features from background particles are also accessible.

A further improvement can be envisaged in the application of methods of machine learning (namely, Convolutional Neural Networks) to the images, or intermediate visual analysis data for a reconstruction of smoother and more reliable profiles.

## Data Availability

All requests on the availability of experimental data should be addressed to the ESA Space Grains topical team coordinators (www.spacegrains.org).
